# Des Anévrysmes Et De Leur Traitement

**Published:** 1857-05

**Authors:** 


NORTH AMERICAN

MEDICO-CHIRURGICAL REVIEW.

MAY, 1857.

### Analytical and Critical Reviews.

ART. I.—*Des Anévrysmes et de leur Traitement.* Par PAUL BROCA, Agrégé à la Faculté de Médecine de Paris, Chirurgien des Hôpitaux, etc. Ouvrage accompagné de figures intercalées dans le texte. Paris, 1856. 8vo. pp. 931.

(Continued from the March No.)

SECOND PART. TREATMENT OF ANEURISMS. At the commencement of his undertaking M. Broca says: "I shall have to fill up more than one historical gap, and I shall have to examine if certain modes of treatment, which empiricism has found, and which empiricism has afterwards abandoned, merit indeed the almost complete forgetfulness in which at the present day they are left. I shall have, moreover, to describe several very modern methods, such as galvano-puncture, and coagulating injections, methods that have not yet found their place in classic books, or that, scarcely indicated *en passant,* have not yet been the object of a didactic description. Finally, and above all, I shall enter into long developments concerning indirect compression, whose history, scarcely sketched in rough outline by my predecessors, has never been written in a complete manner."

The methods he describes are very numerous, and he thus groups them in a natural and scientific order in a table.

I. DIRECT METHODS.

METHODS HAVING FOR THEIR OBJECT THE SUPPRESSION OF THE TUMOR:

1st. The opening of the sac (method of Antyllus).

2d. Extirpation (method of Purmann).

3d. Cauterization.

METHODS HAVING FOR THEIR OBJECT THE MODIFICATION OF THE TUMOR:

1st. Styptics.
2d. Moxas (method of Larrey).
3d. Endermic method.
4th. Acupuncture (method of Velpeau).
5th. Harelip suture (method of Malgaigne).
6th. Malaxation (method of Fergusson).
7th. Application of heat (method of Everard Home).
8th. Application of refrigerants.
9th. Direct compression.
10th. Galvano-puncture (method of Guérard and Pravaz).
11th. Coagulating injections (method of Monteggia).

II. INDIRECT METHODS.

1st. Medical treatment (method of Valsalva).
2d. Ligature above the sac (method of Anel).
3d. Ligature below the sac (method of Brasdor).
4th. Ligature above and below the sac.
5th. Indirect compression.

III. AMPUTATION.

It will probably strike every one, on the perusal of this table, that in "filling up more than one historical gap," so as to find the true author of a method, a tendency is manifested to allow the names of his own countrymen to push out more conspicuously than any others.

The method of treating aneurism by *opening the sac*, is justly called the *old method*. As it was supposed that the gravity of aneurism arose from the deleterious qualities of the blood accumulated in the sac, in its treatment, the object was to rid the tumor of the blood contained therein. Hence arose the opening of the sac, the method generally known as the *method of Ætius*, as he is the first author by whom it is known to have been described. Ætius was a compiler of the sixth century, and his observations on "a dilatation of the vessels which the Greeks call aneurism," can be seen in Erichsen's Selections, the work before referred to. To attribute to him, however, the old method of treating aneurism is, according to M. Broca, an error which for the future he hopes no one will commit; (p. 207.) It appears from his account that it belongs to Antyllus, a surgeon, who "must have lived before the reign of Julian, called the Apostate, since Oribasus, the physician and friend of this great Emperor, has preserved several chapters of his works." Now, the XLV book of Oribasus, long considered as lost, was found twenty-five years ago, among some manuscripts in the Vatican, by M. Angelo Mai, and it has since been printed in Rome in the original Greek. The XX Chapter of this book is entitled, "On Aneurysm, extracted from Antyllus, his First Book of Surgery." M.

Broca gives the whole chapter. The fundamental idea of this surgeon was to evacuate the blood contained in the tumor, and it led him to institute the great method of opening the sac, and to avert the pressing danger of hemorrhage, a double ligature, applied, previously to the incision, above and below the sac, appeared to him to be indispensable. From this epoch down to the 17th century, there was no modification in the method of treating aneurism; at this time, the invention of the tourniquet, made by Morel, at the siege of Besançon, in 1674, by furnishing a means of arresting hemorrhage in the course of operations, gave rise to a very important modification, and allowed the operation, which on account of the difficulty of previously finding the arterial extremities, had been inaccessible to most surgeons, to become the usual practice. The surgeon could now first open the tumor, and this manœuvre, now free from danger, facilitated greatly the search for and ligature of the two ends of the artery. This method of operating for aneurism was the one generally adopted until 1785, when Hunter and Desault introduced another method, created by Anel, in 1710, but which had been neglected by most surgeons.

The next Chapter is devoted to the *double ligature*, or ligature above and below the sac, a method of treatment, to which, in his table, he assigns a different place. He believes that it was first employed by Pasquier, in September, 1812. There is no advantage whatever in cases of arterial aneurism, in the double ligature, and, on the contrary, many inconveniences in leaving in the sac an inorganizable mass of blood, that could only provoke inflammation. In varicose aneurism, however, when the blood could easily pass from the artery into the vein, the accidents resulting from the pressure of passive clots are not to be dreaded, and in the exceptional cases where varicose aneurisms must be operated upon with the knife, recourse should be had to this plan. M. Broca recommends Malgaigne's method of making two small incisions above and below the tumor, in place of a long one over its whole length.

*Extirpation* of the aneurismal tumor, called the method of Purmann, "who re-imagined the operation of the ancients condemned by Antyllus, and gave it his name," (p. 219), has been very rarely performed, and when it has been, generally by mistake, the surgeon mistaking the tumor for one of a cancerous or fibrous character. It is more serious and more difficult than opening the sac, which itself is inferior to most other methods.

M. Broca commences Chapter IV, on *cauterization*, thus: "Sprengel, who has written on the history of the treatment of aneurisms, a chapter where there are more errors than lines, pretends," &c. This is a mild accusation; in the edition we have seen this chapter contains seventeen octavo pages. "For this, the actual cautery and caustics have been used." "The actual cautery is only applicable when the tumor is very small and very superficial." "It is necessary to destroy the whole tumor, and to reach directly the artery, a thing often impossible, and always doubtful." "Whenever caustics have been made use of, the sac has been opened before the artery has been obliterated, even when the chloride of zinc was used, whose coagulating properties are very great." "This faithless method of cauterization should be almost entirely banished from practice; it can only be applied with security on the aneurisms of very small arteries, or at least on those that can be controlled permanently by means of a tourniquet or garrot placed above the sac, and for these cases many other modes more efficacious are in our possession;" (p. 226.) When the vessel is one of the large arterial trunks, and is so near the trunk of the body that the method of Anel is inapplicable, M. Broca says that he does not "dare to pronounce absolutely" that he would not use caustics, but that he would only have recourse to this method at the last extremity, and after having determined the inefficacy of galvano-puncture and coagulating injections; (p. 228.) We have no doubt that as M. Broca, in such cases, intends to use previously those methods, he will never have occasion to have recourse to the application of caustics upon the tumor, but we wish to exclaim against the principle, that guides M. Broca in all cases; namely, that the surgeon must do something. He might have learned from M. Nélaton, for whom he deservedly manifests great esteem, that in surgery, "il faut savoir se resigner," that the surgeon must know how to resign himself not to interfere when all interference must be dangerous. How M. Broca, after expressing, and with truth, we believe, such ideas as we have given above, translated from his work, can shortly afterward express hesitation as to whether he would not apply caustics upon an aneurism of a large trunk, situated near the trunk of the body, we do not understand. Any one, however, who would be inclined to listen to him so far, would in all probability follow him implicitly, and after coagulating injections and galvano-puncture, the patient is not likely to be harmed by the caustics.

*Styptics* or *astringents* have been applied to the skin covering the aneurism, with the object of giving to the walls of the artery the tenacity they have lost. This method of treatment is very ancient, and very inefficacious.

The application of *moxas* (method of Larrey), over the aneurism must be proscribed. It has been pretended that an inflammation would be propagated to the walls of the sac, by which the coagulation of the blood would be brought about; but this effect is very doubtful, and it is dangerous to alter the continuity of the skin covering the aneurism.

M. Thierry, and after him, many others, having previously denuded the dermis by the application of a blister, succeeded in coagulating, by applications of the perchloride of iron, the blood in enlarged veins. M. Broca, supposing that the perchloride could as well reach the arteries as the veins, thus cured an arterial varix of the scalp, and reports the case at length. It has never yet been applied in the treatment of aneurisms, properly so called, but he proposes to treat in this way "all aneurisms of the scalp and frontal region, and generally all aneurisms at once not voluminous and very superficial;" (p. 233.) It probably acts by provoking an inflammation which is propagated to their walls, for in the case of arterial varix the obliteration was only effected in five days after the application. "It acts by the production of passive clots, but they can only be injurious when they form a large mass, and their presence is without danger in small aneurysms." No name has been attached to this method, which may with justice be called, *the method of Broca;* it can only be his modesty, that has prevented him from taking what is justly his due.

*Acupuncture, Velpeau's method*, has been seldom employed in the treatment of aneurism. The paper of M. Velpeau on the subject is in the Archives Générales, for 1831. He says: "He hoped not only that the vessel would be obliterated, but that it would be gradually obliterated,—a result vainly sought after by surgeons to this day. By thus insensibly closing up, the circulation would have time to seek new channels. Preserving their natural relations, remaining protected by the surrounding tissues, scarcely injured in their structure, the fear of consecutive hemorrhage would be less founded than after the ligature." (Loc. cit. p. 283.) This plan presents itself under theoretical appearances that render it very worthy of interest. When M. Velpeau left an acupuncture needle in the artery of a dog, a clot formed around it and the vessel was obliterated. Upon man, however, this desired result has never taken place, in the few cases in which it has been tried. The application of the needles to the sac itself, is the only one that M. Broca studies. "One needle is not sufficient to close the sac, and by using more, the chances of inflammation of the sac increase at the same time as those of fibrinous coagulation;" (p. 236.) "Acupuncture might do very well for small aneurisms, but it is not for small aneurisms that new methods are demanded; they yield to a great number, that only leave, so to speak, l'embarras de choix. What surgery seeks is a means capable of curing the aneurisms of large arteries without danger, and experience has already shown that in order to accomplish this end, acupuncture must not be counted upon;" (p. 239.)

In *Malgaigne's method* of treating aneurism, which is only applicable to small and superficial arteries, needles are passed through the tumor, and then a thread is passed around them as in a suture for hare-lip. (Malgaigne, Manuel de Med. Op. 6th ed. Paris, 1853, p. 203.) To the action exercised by the foreign body left in the tumor, is added the stricture produced by the thread. The greater part of the cavity is thus effaced, and the rest is inflamed and fills with clots. " Only suitable for small and superficial arteries, and for these there are other methods as inoffensive, whose efficiency experience has demonstrated."

*Malaxation* was imagined by Fergusson, for the purpose of breaking up and detaching pieces of the thick layer of coagulated fibrine lining the sac, which should be carried by the current into the lower end of the artery and stop it up. Two cases thus treated have been reported, one first published in the Medical Times and Gazette (Vol. I, p. 255), can be seen in the Medical Examiner of Philadelphia, for June, 1852. The termination of this case is not given in the journals, but it appears, " that the patient was doing well, and the tumor gradually diminishing, when, four months after the manipulations, the patient was attacked with so violent pain in the middle of the tumor, that he died from it in three days." The autopsy made it probable that the pain was owing to pressure on the brachial plexus, on which side the tumor had suddenly increased in size; (p. 244.) The second case, also by Mr. Fergusson, is reported in the London Lancet, for September, 1855, and also in the American Journal of Med. Science, for the following October. The Lancet "purposely abstains from giving the case in detail, as Mr. Fergusson expressed his intention of bringing the entire subject under the notice of the Medico-Chirurgical Society." We shall be glad to hear more of this method, of which we are inclined to have a more favorable opinion than the one given by M. Broca. He says that it is very uncertain and very dangerous, and that he " would always give the preference to one of those methods whose value has already been proved, such as galvano-puncture or coagulating injections, or even the method of Brasdor, of which malaxation is only a dangerous modification;" (p. 245.)

We do not think the method to be so very uncertain, or so very dangerous, as the methods to which M. Broca gives the preference. Dr. Carte, who has had great opportunities for observing the various phenomena which arise during the cure of aneurism (indirect compression is spoken of), believes that many, if not all of the rapid cures are effected by a loose clot blocking up the distal portion of the artery. (Dublin Quarterly Jour. of Med. for November, 1854, p. 320.) Dr. Colles, at the same time says, " that we can scarcely conceive it possible, that a cure which occupies 7, 10, or even 30 or 40 hours, could be effected by the slow process of a deposit, layer by layer, until the sac be filled. We see, also, that a similar result has been obtained purposely by the manipulation of the sac, a process which is too dangerous to be otherwise than exceptional in its application." For us, the exceptional cases would be those in which the aneurismal sac is so near the trunk of the body that indirect compression cannot be used. We would say here, in passing, that in a report made by Erichsen, on a case in which small fibrinous clots had been carried from one part of the circulating current to another, he says, "that in every instance which has fallen under his observation, pains in the limbs, sometimes of an intensely severe character, and occasionally associated with an exquisite degree of cutaneous sensibility, have been experienced." (Transactions of Path. Soc., London, 1856, p. 173.) In all cases undergoing indirect compression, in which this peculiar pain is suddenly felt, may we not believe it to be owing to the *sudden* arrest of the circulation in the artery on the distal side, and by a detached clot?

*Application of heat* is called the method of Everard Home, by M. Broca. It is attributed, the suggestion of it at least, to Monteggio, of Milan, in the work of Bellingham; (loc. cit. p. 98.) It exposes to prompt relapses and all the dangers of primitive and consecutive inflammation; (p. 249.)

*Refrigerants* having been used for ages in the treatment of aneurisms, M. Broca occupies a proportionate quantity of his own time in rectifying historical errors. He also speaks at great length of their action, which, whenever they have acted at all on the contents of the aneurism, has been the formation of passive clots. The following remarks are very just: "Many surgeons, counting on their harmlessness at least, have been in the habit of associating refrigerants with other methods, above all with indirect compression. Now, this is a serious practical error, for not only they do not aid, but they act in a contrary manner: one causes active clots to be deposited, the other passive;" (p. 263.) He advises the use of refrigerants in inflamed aneurismal tumor, and thinks that they may be a very useful resource, when the seat of the tumor opposes every operation, combined with the method of Valsalva; (p. 275.)

*Direct Compression.* Very different methods in which compressive means are had recourse to, bear the common name of *treatment by compression*, and it is a source of a great deal of embarrassment to one making bibliographical researches on the subject.

*Mediate* compression applied on the surface of the skin, must be distinguished from *immediate*, which is applied in the bottom of a wound or of a previous incision. Immediate compression can be applied on the aneurism itself, or on the artery at some distance; it can be, in other words, direct or indirect. Immediate compression, however, direct or indirect, is no longer called compression; it is made to enter in the two methods of ligature and of opening of the sac. Fifty years ago, however, it was not so, and any one who was ignorant of this, would be exposed to commit many bibliographical mistakes.

By *compression*, M. Broca means, simply, mediate compression. It comprehends three methods, *direct*, *indirect*, and *general compression of the*

*limb.* Direct, is on the tumor; indirect, above or below the tumor. *General compression* of the limb there is no necessity for defining. It can be practised in two ways; sometimes a bandage is applied over the whole limb, and it exercises direct pressure on the tumor as it passes over it; at others, it is so applied as to exercise direct compression on the aneurism, and indirect compression on the arterial trunk. This latter is the proceeding of Guattani, nowadays abandoned.

After having given these general explanations, M. Broca enters upon the study of direct compression. As usual, a great deal of time is devoted to historical questions. It seems strange that this was not the first mode of treating a soft, pulsating tumor, but according to M. Broca it was indicated for the first time by the Arabs. We learn from Bellingham (loc. cit. p. 5), that Galen cured an aneurism of the bend of the arm by means of a sponge and bandage, but according to Broca it was not an aneurism, but a recent prick of the humoral artery. Not being able to consult the works of Galen, we leave the question where it stands. Sprengel, in his History of Medicine, a work towards which M. Broca, on all occasions, manifests great antipathy, says that Jean de Vigo, a physician of Genoa, who lived at the beginning of the 16th century, was the first to conceive this plan of treatment. Broca declares that the Genoese physician says not one word about aneurisms. Dezeimeris accorded the priority to the Abbé Bourdelot, who, Bellingham says, was the first to contrive and make use of a compressing instrument for the cure of aneurism. The letter of Bourdelot (1681) is to be found in Erichsen (Observations, &c., p. 207). Porter, in his valuable work, has made the mistake of supposing it to be the celebrated Bourdéloue, at which M. Broca expresses admiration. According to M. Broca, instruments like those used for hernia, were employed by the Arabs. At all events, direct compression was long used, and many instruments were invented, but, in spite of them, it could not have furnished many successes, for it was almost abandoned when Guattani modified it in a very important manner, by combining direct compression with indirect; and this will be studied elsewhere. The edition of Guattani that we have seen is in Lauth's collection of Latin writers on aneurisms, Strasburg, 1785. Lauth takes from the edition published in Rome in 1772, and says, page 5, "Quæ externis aneurysmatibus ferendæ curationes manuales sint, tentaminibus multis atque multifariis, edocuit Guattani."

M. Broca studies at great length the effects and the mode of action of direct compression, in order to see if it merits the neglect into which it has fallen. It has been employed as palliative, in order to arrest or retard the development of the aneurism; and as curative. When employed in the former way, "it effectively opposes the increase of the sac towards the surface of the limb, but the tumor increases in other directions. Besides, it prevents the skin from yielding to the pulsations of the sac; it accelerates the adhesion of the sac to the skin, and seems to provoke the rupture of the aneurism externally." He does not hesitate to reject it entirely. When employed as *curative* it presents several other inconveniences. When the aneurism is small, regular, superficial, and near the bone, a moderate pressure is sufficient to reduce it. Aneurisms at the elbow, when commencing, present these favorable conditions, and the compression can almost always be supported without difficulty. But when the aneurism is large and deep-seated, the pressure must be very considerable to prevent the blood from entering the sac. Very often the pain is intolerable; the skin has a great tendency to inflame and become gangrenous; the collateral circulation is greatly impeded, and gangrene of the inferior portion of the limb is liable to take place. In spite of these unfavorable conditions, however, some patients have been cured by direct compression, and it is interesting, therefore, to study the mechanism by which it acts.

Direct compression was supposed by Guattani to owe its superiority to its leaving the artery permeable; a circumstance supposed to be favorable against the occurrence of gangrene of the limb. Pathological anatomy, however, soon showed, that, contrary to all received theories, direct compression most generally caused the obliteration of the arterial trunk at the place of the aneurism. From one excess surgeons went to another, and it was pretended by Scarpa, as may be seen from the citation given above, that no aneurism could get well without the complete obliteration of the corresponding artery, and its transformation into a ligamentous cord. Scarpa does not deny that direct pressure can cure the aneurism, but he says the pressure must be carried so far as to produce adhesive inflammation of the proper tunics of the artery. M. Broca explains the diversity in the theories by the diversity of the real results, and allows to this method three very different methods of action: "sometimes it obliterates the tumor by passive clots; sometimes it causes the deposition of active clots; and, finally, it causes the tumor to retrograde slowly, and the aneurism is slowly cured by a special mechanism;" (p. 279.) The obliteration by passive clots is generally due to an inflammation caused by the bandage, which follows the same march as spontaneous inflammations, already studied. The second mode of cure is more rare, and M. Broca believes is always owing to some other method made use of at the same time, as that of Valsalva, or of indirect compression. Moreover, he believes, that in these cases, these methods have produced the deposition of fibrinous layers, in spite of the direct compression, "for it prevents the circulation of blood in the aneurism, and hence opposes the gradual deposition of the fibrine;" (p. 282.) It is irrational, therefore, in his view, to join this method to that of indirect compression, as many surgeons have done. In many cases it is impossible to refuse to admit that it has hastened the cure after indirect compression had been used for some time; in such, he says, that the tumor is too hard to be affected by the compression, which is, therefore, transmitted to the arterial trunk, in which the circulation is thereby diminished; (p. 283.)

The third mode of cure is much more common than the others, and is entirely special. The tumor, without ceasing to pulsate, becomes gradually smaller, and the aneurism disappears little by little. It is rather difficult, according to M. Broca, to understand this cure, for no matter how small the aneurism may be, unless the artery be obliterated, or the orifice of the sac at least, the cure could not be effected. Sometimes the artery is not obliterated, and when so, the agent of cure must be the closure of the orifice of the sac. Autopsies never show the edges of the orifice adherent; they are always separated by a small *bouchon*, very resisting, as solid as a cicatrix. This *bouchon* is continuous with a small solid mass of the same substance, situated in the place of the old sac. This, he says, would seem to indicate that obliteration had taken place by means of fibrinous clots; but if so, the tumor should have ceased to pulsate at a time when it was still of a certain size, and instead of that it preserved its pulsations to the last. He accounts for these phenomena by supposing that in all these cases, a layer of fibrine originally lined the sac, and that the compression gradually pushed it towards the orifice, and finally blocked it up. We ourselves believe, in accordance with the views we have expressed before, that this is erroneous, and that direct compression acts simply by the mechanical support it affords the sac. The process by which aneurisms are generally cured takes place in their walls themselves, and it is aided either by supporting them against the force of the blood, by direct compression, or by diminishing the force of the blood, by indirect compression. "At the bend of the arm the artery is superficial, the tumor is recognized very soon, the bone is near, and the osseous projections protect the collateral arteries, so that direct compression is wonderfully suited to such aneurisms." Aneurisms here are often varicose, and those which escape the action of most therapeutic means, are readily cured by direct compression. M. Nélaton has studied the succession of phenomena occurring in such cases, and he shows that the "double-current bellows-sound," and the peculiar thrill disappear while the tumor is still continuing to pulsate, showing that the varicose aneurism is first transformed into simple arterial aneurism, which is then cured.

The recapitulation of the principal points in the history of direct compression, is thus made by M. Broca. "We find that this method is always very uncertain in its results; that it is completely inefficacious in the greater number of cases; that it is often intolerable; that it exposes to inflammation, to rupture of the sac, to gangrene of the tumor, and to gangrene of the limb; that it ought to be absolutely banished from the treatment of voluminous aneurisms; and that it appears to be reserved only for small and recent traumatic aneurisms of the elbow;" (p. 298.) The light in which he regards this mode of treatment is certainly not the most favorable, but it is, at least, consistent with his views on the subject of the cure of aneurism, and we wonder therefore that it is so often employed by surgeons who hold the same opinions. May not this general employment, conclusive evidence of its value, notwithstanding what M. Broca says, be taken as strong proof, indirect though it be, of the erroneousness of these opinions?

*Galvano-puncture* (*Method of Guérard and Pravaz*).—As the result of his historical researches on this mode of treating aneurism, M. Broca has found, that the first idea belongs to M. Alphonse Guérard (1831); that the first experiments on animals were made by Guérard and Pravaz; that the first attempt on man was made by an anonymous surgeon in Paris, cited in a thesis, in 1837; and finally, that the first success belongs to M. Petrequin, in 1845. In this historical gap, Mr. Phillips and Dr. O'Shaughnessy have completely disappeared.

As galvano-puncture acts by producing a mass of coagulum, and also by producing inflammation of the walls of the sac, we are amazed to hear "that everything permits me to believe that ulterior researches, by perfecting its application, will render it at once more efficacious and less dangerous, and I would not be surprised that, sooner or later, it should become superior to the ligature; at the present day already, it is almost capable of entering into parallel with it; it has, moreover, the advantage of being applicable to certain aneurisms inaccessible to most other methods." This last advantage amounts simply to this: that it enables a surgeon to do something; whether that something or not be beneficial, is not the question, but he must be enabled to do something, he must have something *applicable*.

As the results of galvano-puncture have often been fearful, and as M. Broca, for some reason or other not very evident to us, is determined to advise its use, over seventy pages of his work are occupied in treating the subject. A great number of these were necessary in order to come to the historical results which we have given; others are occupied with his experiments on the coagulation of blood; others in a dissertation on galvanic piles; tension and intensity of electricity, occupy others; in short, it is an exceedingly tiresome chapter. The following points are those of most importance:—Galvanic clots do not possess any of the qualities of active clots. Very often, at the expiration of a few hours, they are broken up and carried away by the current of the circulation. When they remain they frequently give rise to consecutive inflammation, to suppuration and gangrene; and when an opportunity is given for their examination, they are found to be constituted of a soft and homogeneous substance, absolutely different from that constituting active clots. The analogy between galvanic clots and ordinary passive clots cannot then be misunderstood. This analogy, however, does not go so far as identity. Passive clots are composed of a mass of globules imprisoned in a network of fibrine. The greater part of the galvanic clot is likewise formed by the globules, and there is every reason for believing that a certain quantity of fibrine is also solidified; but there is a third element, which constitutes the distinctive character of the galvanic clot,—it is the coagulated albumen. M. Broca insists, at great length, and with force, on the refutation of the theory that galvanism acts by actual cauterization, and he shows that the scars in the skin are owing to the diffusion of the electrical fluid in the tissues traversed by the needles. As he believes that a want of perfection in the mode of operating has been the cause of failure, he insists much upon its details. He gives the preference to needles, varnished in the middle, about $\frac{1}{75}$th of an inch in diameter, and a great number of these needles are to be attached to each pole. Immediate coagulation must be sought for; and, whenever it is possible, compression should be made, in order to diminish the force of the current of the blood. The action of the currents must never be interrupted. The first galvanization should always be made with feeble currents, and at the following sittings the force should be gradually augmented.

The operation is very painful, it exposes to many serious accidents, and the effect, when obliteration is produced, is the production of a mass of clots that are generally carried away by the current, and, when they remain, act as passive clots. "Not only," says Erichsen, "is the principle on which it is attempted to procure obliteration of the sac in galvano-puncture a vicious and peculiarly dangerous one, viz., by the coagulation of the blood, and the inflammation of the wall of the sac, but the results that have hitherto been obtained by this method are not such as would justify a prudent surgeon, in submitting his patient to experiments of this kind, when he possesses so certain and comparatively a safe mode of cure as that by deligation or compression." (Loc. cit. p. 474.)

*Coagulating Injections* (method of Monteggia).—"Mr. Wardrop has suggested the injection of dilute acetic acid into the sac, with the view of causing the formation of a coagulum," says Bellingham (loc. cit. p. 114). But he does so, in the article he has written on aneurism in the Cyclopædia of Practical Surgery, after citing the proposition of Monteggia. At the commencement of 1853, Pravaz used the perchloride of iron, and in the month of November, when Malgaigne read a communication to the

Academy on the subject, already 11 persons had been operated upon, resulting in 4 deaths, 5 serious reverses, 2 cures. Moreover, these two cures were obtained "at the price of such accidents, and with doses so exaggerated, that in place of praising the operators, he blames their imprudence." The discussion may be seen in the Arch. Gen., 5th series, vol. 2, p. 741. M. Broca devotes fifty-two pages of his work to this method of treatment, where the mode of operating is described very minutely. The dangers of this method are very great, and there is no known way of avoiding them. Every liquid that gives rise to very prompt coagulation of the blood, and to a very solid clot, has a very irritating action on the tissues of the body, and it is much to be doubted if any will ever be found that has not. The perchloride of iron produces a very firm clot, and there is no example where the pulsations have reappeared, when once entirely suppressed. It is dangerous on account of its irritating action, and on account of the consecutive inflammation.

It is indispensable to withdraw the tumor, during the whole operation, from the shock of the blood, for the action of the perchloride is never instantaneous. In all experiments, in which the blood withdrawn from the body was mixed by means of a glass rod with the perchloride, at least thirty seconds elapsed before the coagulation of the blood; and thirty seconds represent thirty arterial pulsations: hence the blood in the sac would be renewed, and the perchloride carried away. It would be folly therefore to attempt to throw the injection into an aneurism, inaccessible to Anel's method.

This solution of the sesquichloride of iron, called perchloride by the French, is not the Tincture of the Chloride of Iron of the United States Dispensatory, as is stated in that work. It is a solution of much greater strength, prepared in a peculiar manner.

M. Broca has here finished the study of the direct methods of treatment, and commences the indirect, or those in which the aneurism is acted upon only by the intermedium of the circulation. The first he considers is the *Method of Valsalva*, the name which is given to the *ensemble* of means, that have for their object to diminish the energy of the general circulation. The idea of bleeding persons affected with aneurism is certainly anterior to Valsalva, but he made diet and bloodletting a regular method, which he applied several times with complete success. He prescribed bleedings, repeated at short intervals, an absolute repose in bed, complete tranquillity, and a regimen of truly frightful severity. By this treatment, undoubted cures have resulted, and as Hodgson has shown, these cures are entirely similar to natural spontaneous cures, or those that result from fibrinous depositions; which he, as most authors, believes to come from the circulating blood. Hodgson thus ends that portion of his work devoted to the spontaneous cure and medical treatment of aneurisms. "The deposition of coagulum in the cavity of the aneurismal sac and the artery leading into it, is the mode by which the spontaneous cure of aneurism is, in most cases, effected. The formation of coagulum being a general occurrence in aneurisms, it is an important object to prevent the increase of the sac, that the deposition of the coagulum may proceed to such an extent as to obliterate the cavity. It is the force of the circulation which causes the enlargement of the sac, and its ultimate rupture; hence the diminution of the force of the circulation is the principal indication in promoting the cure of aneurisms." (Loc. cit. p. 163.) In a purely pathologico-physiological point of view the method of Valsalva is superior to every other, unless it be indirect compression, but in a practical point of view it is the contrary. Many patients cannot support it, and in many cases the advantageous effects of the depletion produced by the bloodletting are neutralized, and more than neutralized, by the effect it produces on the blood. Dupuytren says, " that he has remarked that it diminishes the resistance of the walls of the aneurism more than it enfeebles the heart's action; and further, that when the surgeon, tired of trying this mode of treatment ineffectually, begins to supply the patient with nourishment, with a view of operating, the tumor, surrounded by parts which have lost their elasticity, advances rapidly under the influence of the *vis à tergo*." (Loc. cit. p. 29.) M. Broca says, "The method of Valsalva, properly so called, should only be put into use in desperate cases, when the elements comprising it render important services in a great number of cases, when they are employed with moderation, and they ought consequently to be always present in the mind of the surgeon." (Page 436.)

The blood in the arteries circulates under the influence of two principal causes, pressure and impulse; the former from the arteries, the latter from the heart. The force of the impulse of the heart varies in different animals; the pressure is always about the same: it is about 120 millimeters, that is, it is capable of raising a column of mercury to that height. In a rabbit, every time the heart beats, the column is raised 5 millimeters more, in a dog 12, in a horse 40. It is thus seen that though the force of the impulse varies, it is always far less than that of the arterial pressure. The force by which an aneurism is distended is therefore principally arterial pressure. To show the influence of the quantity of blood upon this force, in one experiment we saw made by M. Bernard, while before the bloodletting the pressure was 141 millimeters, afterwards it was but 57. What an enormous reduction can thus be effected in the force with which the blood distends an aneurismal sac! At the same time, however, the impulse of the heart is much augmented; and the effect of bleeding upon the blood is unfavorable to the production of those changes in the walls of the sac, which we believe lead to cure. The experiments of M. Bernard show that some substances influence the arterial pressure otherwise than by mere quantity, that is, by merely influencing the bulk of the circulating liquid. Coffee does so to a very great degree, and therefore should be strictly interdicted. The respiration of the patient should be made as free as possible, for the arterial pressure is thus very much diminished. In expiration there is constantly an augmentation of pressure, which a cough or sneeze produces very suddenly, so that it is not rare at such moments to see an aneurism burst. The influence of the nerves is also very marked, as some experiments made with galvanism show, but this action is out of our control. There are a multitude of considerations on the causes influencing the general circulation; all of which are concerned more or less in the treatment of aneurism by the method of Valsalva. This chapter, which M. Broca might have made one of the most interesting and instructive in his whole volume, is singularly the contrary. We make, however, the following extract from it, as illustrative of his style. *Le style, c'est l'homme.* "Let us pardon the great Morgagni, who was however the pupil and friend of Valsalva, for having been able to believe that one of the writers known under the name of Hippocrates had been the inventor of this method. The Hippocratic legion did not even know how to distinguish arteries from veins, and consequently was ignorant of the very existence of aneurism. A total absence of critique, a blind confidence in the omniscience of the ancients, have alone been able to cause the acceptance of the unsustainable opinion of Morgagni. Thus, Hodgson, speaking of the medical treatment of aneurism, says that this practice, suggested by Hippocrates, was confirmed by the experience of Valsalva; and Pelletan himself, after having found by examination that the Hippocratic text had reference to a very different affection from aneurism, does not hesitate to say that Valsalva *must* have found the idea of his method in the writings of Hippocrates. This manner of writing history is very easy. The fact is, that the author of the treatise 'De Morbis,' giving a fantastic description to I know not what disease of the lung, that commences by varix and ends by suppuration, has advised to treat by diet and bleeding the patients, who, by chance, would be attacked with this imaginary disease;" (p. 420.)

The ensuing 440 pages, from page 437 to 877, are devoted to the ligature above the sac, the ligature below the sac, and indirect compression.

The method by ligature above the sac, M. Broca designates as the method of Anel, "as it is just that the name of a man should be attached to the progress that he has realized." As to the *operation* of the ligature upon an arterial trunk, he very properly says nothing about it; it belongs no more to the history of aneurisms, than to that of arterial wounds. We will give a *résumé* of this Chapter, the 17th, for we think it most admirable.

It was Anel, in 1710, who first operated thus for aneurism, upon a case of traumatic aneurism, at the elbow-joint. In the reflections following the history of the case he says: "Instead of placing a ligature above and below the aneurism, I only did so above; moreover, surgeons open the aneurismal sac, and I did not touch it, not doubting that the blood contained in the sac would be dissipated, and that the sac once emptied would not again be filled; that the tunics of the membranes forming it would not fail to diminish, and that thus the tumor would disappear; this did not fail to happen as I had thought." This shows that Anel knew what he was doing, and why he did it; that he was led by his knowledge to give up opening the sac, and thus he created a new method. For three quarters of a century this method was applied only to the aneurisms of rather small arteries. It was in 1785 that it was applied for the first time to the treatment of popliteal aneurism, by Desault. The patient recovered, dying of necrosis of the inferior end of the tibia. In December, 1785, six months after Desault, John Hunter operated for popliteal aneurism. Instead of searching for the popliteal artery, Hunter tied the femoral before it passes through the sheath of the adductor muscle. His object in so doing was to find a spot where the artery would certainly be healthy; believing that the obliteration provoked by the ligature was the result of union by first intention, and that this adhesive process was only possible in arterial walls free from all alteration. He believed that by tying the artery at a distance from the aneurism, that he would avoid the section of the arterial tunics, and the production of consecutive hemorrhages, which, however, experience has shown to be as frequent after his mode of operating as after that of Anel and Desault. All the theoretical reasons by which Hunter was led to leave, a little, the way of his predecessors, are false. Hunter invented a useful proceeding, but he did not create a new method. His first patient dying fifteen months after the operation, he afterwards found, at the autopsy, that the ligature acted simply by causing solidification of the blood, and although he drew no distinction between the two kinds of clot, this discovery was a true progress. There is no reason why this method should receive the name of the Method of Hunter. The idea was not his, of replacing, by a simple ligature applied above the sac, the cruel operation of the ancients; this idea belongs to Anel. He is not the first who put this idea into execution. He was not the first to attack the great arteries by it. He did not have the first case of success. Finally, it was not Hunter who discovered the mode in which the ligature acts upon the walls of the artery. It may be said that, by applying the ligature at a distance from the sac, that he found the best proceeding, the best way of performing the operation, but the proceeding of Scarpa has received the preference at the present day, and should this method be called the method of Scarpa, until another surgeon shall have shown the advantages of another place for the application of the ligature? M. Broca says: "Far from me the thought of seeking to diminish the glory of the great Hunter, whose indefatigable genius has exercised so great an influence on the destinies of science. England is justly proud of having produced this surprising man, who I consider, for my part, as the principal creator of pathological physiology;" (p. 464.) At all events the labors of Hunter have caused the adoption in general practice of the method by ligature. Its triumph was legitimate, for compared with the other methods, it must be considered as excellent. The hopes it had aroused, however, have not been realized; statistics show that it is one of the most serious operations in surgery; that it is followed by the death of the patient in nearly one case out of three. These statistics are cited further on; the mode in which the ligature acts and the causes of the accidents it produces are first studied.

The method of Anel can be applied in two very different ways: the ligature may be tied immediately above the sac, according to the proceeding of Anel, or at some distance, according to the proceeding of Hunter. Each of the proceedings has its respective advantages and inconveniences. The real difference between them, however, is not that maintained by the schools of Hunter and of Scarpa. Whatever may have been said, the structure of the walls of the arteries is almost always unaltered in the neighborhood of external aneurisms. When it is otherwise no one can tell how far the alteration extends, and wherever he places the ligature, the surgeon is liable to fall upon a diseased vessel. When the arteries are diseased the cellular coat is almost always intact, and what difference does it make if the others be more brittle than they are normally, since it is desired to divide them at once? If the proceedings differ in some respects, it is by their action on the aneurismal sac, and on the general circulation of the limb.

In Anel's proceeding there is no collateral between the ligature and the sac; in that of Hunter, on the contrary, the portion of the artery comprised between these two limits gives off one or several branches of various sizes, capable of bringing again into the aneurism a certain quantity of blood by means of anastomoses. The distance between the sac and the ligature does not, by itself, exert any influence either on the tumor or on the general circulation of the limb. Everything depends upon the presence or the absence of intermediary collaterals, their number, their size, and their connections. Therefore, without paying any attention to the spot where the ligatures are placed, the applications of the method of Anel are divided by M. Broca into two categories, those that leave, and those that do not leave any collateral between the aneurism and the constricting thread. All operations of the first category are brought by him into the proceeding of Anel; all of the seeond, into that of Hunter. It may be objected to this, that these two designations have been turned from their primitive designation, and that the question of intermediary collaterals did not preoccupy either Anel or Hunter. The question is not, however, what these two surgeons intended, but what they did, and classing the proceedings after their mode of action, M. Broca is certainly authorized in his nomenclature.

The effects and mode of action of the method of Anel, that is to say the modifications it causes, in the artery tied, and in the neighboring parts, in the general circulation in the limb, and in the tumor itself, are treated with great ability. The effects on the whole economy, the fever, the nervous irritation, and the visceral congestions, belong to the history of the ligature in general, and offer nothing particular when the ligature is applied to the treatment of aneurism. The direct effects upon the artery offer nothing special, and, in accordance with the views which he has already given, M. Broca does not study them here. The fall of the ligature is often preceded, accompanied, or followed by hemorrhages, called consecutive, furnished either by the superior or the inferior end. Phlebitis is much more common than most authors believe. Another accident much less known, of which M. Broca gives a remarkable example (p. 479), is nevritis. Purulent collections take place sometimes in the common sheath of the vessels. These are the special accidents provoked by the ligature; to this all the ordinary accidents of wounds, erysipelas, diffused phlegmon, tetanus, &c., must be added. "It is but a trifling merit," says Dupuytren, speaking of the application of a ligature for aneurism, "it is but a trifling merit to acquit one's self well in this, the mechanical part of our profession; but the most consummate art is insufficient to insure success and guard against accidental circumstances, which may arise in the after treatment." (Loc. cit. p. 13.)

In the circulation and the nutrition of the limb, the phenomena caused by the action of the method of Anel are proportional to the importance of the vessel tied; and in their study, M. Broca takes as a type, the ligature of their principal artery. When the flow of the blood is arrested therein, it passes by collateral ways, which are of two orders, the arterial collaterals, and the innumerable capillary communications, which form in all vascular parts of the body, and, above all, in the skin, a vast uninterrupted network.

At the moment of tying the artery, the arterial system of the limb, suddenly removed from the influence of the heart, ceases to pulsate, and empties itself into the veins. Most generally the skin becomes pale, and if left exposed to the air, it becomes, in a few moments, very appreciably colder. As a general rule, the cooling of the limb is but temporary, and, at the expiration of one or two hours, gives place to an elevation of temperature, which may be even 5°, 6°, or 7°. This phenomenon at first appears strange, and must be inexplicable to those who concentrate their whole attention upon the arterial anastomoses. It is owing to the capillary anastomoses, which form so rich a network in the skin. The blood enters these capillaries in a greater quantity than usual, and the skin receiving a greater quantity of blood than is normally destined to it, becomes hotter than on the opposite side. There is another cause for this: the constriction of the artery inevitably produces that of the filaments of the sympathetic nerve accompanying it, and the curious phenomena, discovered by M. Bernard, attending injury of this nerve, are well known. The explanation of M. Brown Sequard of these phenomena, is the one adopted by M. Broca; we are by no means inclined to agree with him, but this is not the place to discuss the question. When the arterial anastomoses are large and numerous, the circulation is promptly re-established, and the part of the capillaries is feeble and very soon over. The surgeon is tempted to believe that the exaggeration of temperature is a good sign, and that the limb is less exposed to gangrene the warmer it becomes. This is not so, for the exaggeration of temperature shows on the contrary that the profound circulation in the limb is very insufficient. The collateral capillary circulation is a momentary resource, whilst the arterial anastomoses furnish a definite one.

Thanks to these two collateral circulations, gangrene is very rare after the application of a ligature, when applied for hemorrhages, recent wounds, or tumors not aneurismal. Why, then, is it so common when aneurisms are treated by the method of Anel? It is on account of the arterial obliteration produced almost inevitably at the tumor. It is here, above all, that it is necessary to distinguish the proceeding of Anel from that of Hunter. In the former there is no collateral between the aneurism and the ligature, consequently the obliteration due to the ligature and that which is accomplished at the tumor, are confounded in one and the same obliteration. The course of the blood is intercepted from the first collateral above to the first below, just as in a simple ligature. There is very little danger then from gangrene. In the proceeding of Hunter it is different, and things are changed; the chances of gangrene are carried to a maximum. This is clinically demonstrated in the paragraph devoted by Broca to the general results of the ligature; (pp. 591, 611.) This may be known from a few theoretical observations. Take a particular case, for instance a femoral artery tied in the middle of the thigh for popliteal aneurism.

Immediately after the ligature the collateral circulation commences to take place in the midst of very favorable conditions, from the number and size of the anastomoses existing between the deep femoral, and the superior branches of the popliteal. It is now that the obliteration which takes place at the aneurism is effected, an obliteration, consequently, below the origin of the arteries, the superior branches of the popliteal, which have so far been bringing the blood to the limb. A second collateral circulation must be established between these superior branches of the popliteal, arising above the tumor, and those below it. It will easily be imagined that the chances of gangrene are not only doubled, but rendered tenfold greater. Few patients would escape without the resource, unfortunately uncertain, afforded by the capillary anastomoses. In order to render the operation of Hunter less murderous, from the gangrene, several things have been done to which M. Broca pays some attention. Efforts have been made to obliterate the vessel by degrees, by a gradual ligature, applied for the first time by Antoine Dubois. This gradual ligature, properly managed, offers several advantages which are not to be disdained. The ligature is left for but five or six days, so that the wound is soon relieved of the presence of the foreign body, and the ligature is large and temporary, so that the artery is left permeable. The great cause of gangrene being the obliteration at the level of the aneurism, at the moment the organism has just triumphed over the superior obliteration produced by the ligature, the oblation of the ligature restores to the circulation the requisite energy. The superiority of the gradual ligature over the ordinary in the proceeding of Hunter, does not seem to M. Broca contestable. Indirect compression, that is, compression exerted upon the integument, has, however, all the advantages of the gradual ligature without its disadvantages, it is therefore preferable to it, and still more so to the ordinary ligature There is another accident, following the obstacle opposed to the circulation by a ligature: the limb becomes atrophied and powerless. This, which has been attributed by some authors to a defect in innervation, is attributed by M. Broca to defect in the circulation.

The position of the ligature effects most importantly its mode of action on the aneurismal tumor. When a collateral artery exists between it and the sac, the blood will be brought into the artery between the ligature and the sac, and at once retaking its natural direction, it will pass through the tumor exactly as before the operation, only much more slowly. By Anel's proceeding the blood can only enter by the inferior end, and it can only enter into the aneurism by a sort of reflux. The proceeding of Anel will produce passive coagulation, while that of Hunter will more readily obliterate the sac by active clots.

As a general rule the aneurism, collapsed at the moment of ligature, soon again swells; it most generally does not again become pulsating. When it does, it is by no means unfavorable, for the blood is kept in a state of agitation, proper to prevent obliteration by passive clots. Therefore, the method of Anel may act in two ways: by passive clots, which have already been spoken of, and by active. As it is impossible by the ligature to regulate the quantity of blood passing through the tumor, the method of Anel is very uncertain, as Bellingham has clearly pointed out. (Loc. cit. p. 142.) M. Broca then studies these two modes of action of the ligature, and lays great stress upon consecutive hemorrhage, not that which may be furnished by the artery at the level of the ligature, but that resulting from the opening of the sac, inflamed by the passive clots therein contained,—an accident, very formidable, not at all unfrequent, and which most authors have passed over in silence.

The article on secondary pulsations and relapses of arterial aneurism, treated by the method of Anel, is very long. It is necessary to make a distinction between persistence and return of pulsations and relapse. M. Broca seems to think no one before knew that relapse could take place. Bellingham says, "a cure affected by the ligature can only be permanent when it causes the aneurismal sac to be filled up, and the artery to be obliterated at its seat, after the same manner as by compression. If a loose coagulum of blood merely forms in the sac as a result of the ligature, there is danger either of the sac suppurating, or a secondary aneurism may form at the part." (Loc. cit. p. 173.) M. Broca arbitrarily places in the category of relapse, cases where the tumor takes up and preserves *for more than four months*, the character and march of aneurisms, and again divides relapses into two categories,—prompt relapses, and tardy relapses. The first being those which arise when the patient is still under treatment, and the tardy, those which arise afterwards. As to the treatment of relapse, there is much in length; it only amounts to this, try another method.

In the sixth paragraph, devoted to the effect on arterio-venous aneurisms of the method of Anel, there is not much to notice. His theory for explaining the frequency of gangrene is the following. The capillary anastomoses emptying their blood at once into the veins without the intermedium of the artery, the venous system of the limb operated on receives a greater quantity of blood than the arterial, and the blood passes from the vein into the artery at the opening of communication. As the collateral arteries become enlarged, there is, first a flow from the artery into the vein during the arterial diastole, and then from the vein into the artery during the systole, until, finally, there is a continuous and *saccaded* arterio-venous transfusion. This mixed blood, returning again and again into the same limb, without undergoing pulmonary hematosis, is unfit to sustain the nutrition of the limb, and it becomes gangrenous. Again, passive coagulation is prevented by the incessant renewal of the liquid, and active, by the nature of the blood. "It is then, with justice, that the method of Anel is nowadays banished from the treatment of arterio-venous aneurysms;" (p. 591.)

Under the paragraph entitled general results of the method of Anel, by discussing the tables collected by Phillips, Porter, Lisfranc, and above all, by Norris, of Philadelphia, "to whom science owes such important statistics on the results of surgery," M. Broca shows that the method of Anel, though one of the most remarkable progresses of surgery, still, is very deadly. "If now we consider the frequency and gravity of the accidents provoked directly by the application of the ligature,—such as phlebitis, consecutive hemorrhage, and mortification of the limb,—accidents that make it one of the most murderous operations of surgery; if we think that this risky operation has not even the advantage of acting properly on aneurisms, and that, inferior in this respect to any other methods, it very frequently causes passive clots to be deposited in the sac, followed by inflammation, by suppuration, by rupture, by formidable hemorrhages; if we recollect that it exposes to atrophy, to paralysis, to retraction of the limb, the persons it does not kill; if we add, finally, that among the successes attributed to it, figure many patients mutilated by consecutive amputation, and many others, who, after having passed through so many dangerous risks, have had the pain of seeing their disease return,—we arrive at this conclusion, that the method of Anel is dangerous, uncertain, defective, and that, far from constituting the beau ideal of the treatment of aneurisms, it only ought to remain in surgery as a sort of *pis-aller*;" (p. 611.)

Chapter XVIII. (Method of Brasdor.)

The history of the method by ligature below the sac, and of the modifications it has undergone, is done by M. Broca, with great care. He believes, from his researches, that Brasdor instituted the method, that Deschamps first dared to execute the idea, and that Wardrop obtained the first success, and by leaving a collateral between the sac and the ligature, created a new proceeding, which, by extending the sphere of application of the method of Brasdor, has enlarged the boundaries of operative medicine; (p. 626.) The phenomena resulting from the action of the ligature on the artery and on the circulation resemble, in many respects, what has been studied *à propos* of the method of Anel. Gangrene and consecutive hemorrhage he believes to be less frequent than after the method of Anel. That curious problem of pathological physiology, that the aneurism is immediately collapsed when the artery is tied between it and capillaries, he cannot solve. After a repetition of what has been said of active and passive clots, he says, that the only thing which really argues against the operation is the uncertainty of its effect on the aneurism, an uncertainty, which, as he can attribute it to nothing else, he assigns to the individual plasticity of the blood. Of fifteen cases operated upon, according to the proceeding of Wardrop, nine died from the operation; of the six others two were cured, a third was followed by a relapse at the expiration of two years, and in three cases the ligature did not prevent the tumor from making new progress. The proceeding is then very dangerous, and very uncertain, but, as without it death is inevitable, M. Broca concludes that it should be applied to the treatment of certain aneurisms, those of the arteria innominata, and of the internal extremity of the subclavian.

The Lancet for June, 1856, mentions a case, where the right common carotid artery was successfully tied by Dr. Wright, of Montreal, for an aneurism of the innominata involving the roots of the common carotid and subclavian arteries. The ligature caused solidification and reduction of the arterial tumor; the patient, a man seventy years of age, survived the operation nearly three months, dying of hemiplegia. It was the occasion of a paper in the Medical Chronicle for March, 1856, in which Dr. Wright, carefully analyzing the various accounts given of this operation, concludes that there still exists abundant justification to warrant a surgeon in again treating innominatal aneurism by ligature of the right common carotid artery.

The 19th chapter is devoted to indirect compression, that is from page 652 to 877. Indirect compression consists in the application of any mechanical apparatus, destined to compress the artery, either above or below the tumor. The compression below the sac, M. Broca declares is so very unimportant, that he does not study it separately, merely indicating some parts belonging to it. His historical researches on the subject occupy ninety-two pages. Of what Mr. Bellingham has done to elucidate these questions, he says, "Although he has consecrated three-fourths of his work to historical considerations on the subject of indirect compression, the Dublin professor has passed in silence a crowd of documents of greater importance, and has grouped the others quite confusedly." He grants, however, that "in spite of these imperfections, Mr. Bellingham has succeeded in writing a book at once interesting and useful," (p. 654.) The statement that three-fourths of his work have been devoted to historical considerations is a great exaggeration, and it has been a fortnnate thing for M. Broca that it is so; but at the same time he should not complain of Bellingham's historical researches, all comprised in 83 pages, for he has made excellent use of them.

In order to simplify the question as much as possible, M. Broca restricts himself as much as he can to the history of indirect compression, or that exerted on the artery without touching the tumor. He then divides the history into three periods, first the *preparatory* period, "when the compression was only exerted as an aid, and without any very decided object."

This is the *Italian period,* and extends until the end of the eighteenth century. 2d. A period of *creation,* "where the indications and mode of application were clearly established, and where compression employed alone obtained a great number of successes, mingled with still more numerous failures." This is the *French period,* it extends to the year 1842. The third is a *period of application,* "when skilful surgeons, with ingenious patience, put in practice principles established during the third period, and obtained by care and perseverance successes, that have thrown those of the ligature into the shade." This period, he says, deserves the name of *Irish period.* This is a strange division of periods, particularly for one who shows so much indignation on the occasion of the Irish polemics. "They disputed to know who had cured the first patient in that islet, they call Ireland, as if chance could be a title of glory, as if science was not of all lands!" (p. 709.)

He insists long on the first attempts of the art, and the numerous checks that preceded the first official success of indirect compression. This first official success, the first time that indirect compression gave a really surgical success was, he says, in a patient of Eschards, treated in the commencement of the year 1801; the treatment lasted eleven months. This is M. Broca's arrangement of the surgeons, the patients, and the dates, and he speaks thus of the history given by Boyer, who, according to his (Broca's) own account, was one of the surgeons in consultation, who advised the treatment. "The vague souvenir of something that had been related to him, mingled with a souvenir just as vague of another history, which he had read in Lassus, were grouped in the memory of the honest Boyer, around a third fact, which he had seen himself *en passant.*" Science belongs to no country, nor to any political creed, and if Boyer was made baron by an emperor of France, he still was one of her greatest and most reliable surgeons. The same may be said here in regard to Dupuytren, baron by a king of France, who is treated generally almost with contempt by this republican author; Dupuytren, whose dexterity, coolness, boldness, and inventive mind, place him in the highest rank of surgeons. M. Broca is angry with Boyer, because he wishes to have a case treated by indirect compression in France before one of Blizzard, of December, 1801. We can find no date attached by Boyer to his history of this case (Mal. Chir. (1814) tome II, p. 204); which Broca says he dates 1814. Bellingham places the case about the year 1810. This must be wrong, however, for, in the volume, it precedes a case (the one of Michaux) treated in 1804. It may be said here, that Boyer's expressed opinions on the subject of indirect compression, are by no means very consistent. He teaches (loc. cit. p. 131) that, as a general rule, it is not admissible in the treatment of aneurism of the femoral artery, because by pressing against the pubis, you renounce the benefit of the communications given off by the profunda, the only resource for the circulation; and at the middle of the thigh, you would be so near the tumor as to endanger its inflammation. And yet, he teaches, that before the ligature it ought always to be employed, "because if it does not cure it insures its success;" (p. 129.) He also teaches that the compressive means should act with sufficient force to prevent the blood from reaching the tumor.

It was Dupuytren who first showed that in arteries treated by compression, the vessel was not obliterated by adhesion of the walls, nor by coagulation of the blood. As the Irish surgeons fought amongst themselves, at this period, Broca indulges himself in thrusting here and there, now at Todd, now at Bellingham, now at Wilde. He says that he feels himself obliged to relate all this quarrelling; why he is we know not, unless it be to gratify his vanity by showing his acquaintance with it; or he may be instigated by a feeling that in some minds takes the place of gratitude. "The surgical world will learn no doubt with reverential awe, that the patient of the deceased Todd, was cured after that of the deceased Duggan, but that deceased Duggan made use of the apparatus of deceased Todd;" (p.710.) The most important epoch in the history of indirect compression was reached, so M. Broca says, the 25th February, 1825, when Guillier Latouche sustained at Strasburg an inaugural thesis, entitled: "*New way of exercising prolonged mediate compression on the principal arteries of the limbs.*" In this thesis, the author says, or rather an anonymous instigator of the thesis says to the author who repeats, that several tourniquets should be used, and alternately made to press on the vessel. This anonymous inventor of the new proceeding had already treated a patient in the service of M. Gama. In order to find his name, M. Broca vainly read through the theses and journals of that time; he vainly appeals to the souvenirs of surgeons who lived at that epoch; all taught him nothing, until as a *last resource*, he addressed himself to M. Gama, and "penetrated into the retreat where this professor, become philosopher, forgets the injustice of men." After some difficulty, M. Gama finds that these ideas on the effects of compression, and the manner of conducting it, belong to M. Belmas, then at the head of the dissecting rooms of the faculty of Strasburg; but who, as M. Broca informs us in a note, to forget nothing of this interesting history, has long ago abandoned the medical career. *Multiple and alternative compression* is therefore designated by M. Broca under the name of *Proceeding of Belmas.* M. Broca graciously pardons the surgeons of Dublin for their ignorance, in 1843, of an obscure thesis, sustained in Strasburg in 1825, and does not wish to accuse them of the design of robbing M. Belmas of what is his due.

In 1843, commences the period of application, or *Irish period,* as M. Broca calls it,—"The application of the principle of alternate and multiple compression, which Bellingham claims as his own." Nothing can be more modest than the manner in which Mr. Bellingham recounts this history, at page 94, &c., of his book. M. Broca exclaims, "Why must Bellingham have spoiled so good a cause, by refusing to his predecessors the homage due to their efforts and their discoveries?" Surely Bellingham was justified in ignoring what M. Broca had the greatest difficulty in discovering; that a M. Belmas, once a surgeon, suggested something to a M. Gama, once a surgeon also, a method that was once applied in a military school at Strasburg, and unsuccessfully, the ligature being afterwards placed on the vessel. "For us, who have followed step by step the origin and development of the ideas on which nowadays repose the compressive method, we cannot award to M. Bellingham any other invention than that of an apparatus of quite mediocre importance; we mean the compressing weight. Brückner, Eschard, Dubois, Dupuytren, and many others, and above all, M. Belmas, whose precious doctrine Guillier Letouche has transmitted to us, have preceded M. Bellingham;" (p. 738.) While in Ireland, Scotland, England, and America, they were curing aneurisms thus, there was nothing done in France, and the principal reason, according to M. Broca, "is perhaps, that tendency of the French mind, which leads us so often to disdain innovations of foreign origin." "Let us forget," he adds, "in the interest of our patients, that this new progress comes to us from a foreign country; or rather, let us remember, in the interest of truth, that it is really of French creation."

In the paragraph on *the mode of action of indirect compression*, there is nothing to be noticed. He agrees with Bellingham as to the mode of action, namely, that by rendering more slow the flow of the blood in the aneurismatic artery, indirect compression favors the spontaneous and progressive coagulation of the fibrine, and imitates as much as possible the process that leads to the natural cure of aneurism. From a number of autopsies, at very various periods after treatment by indirect compression, M. Broca draws the same conclusions drawn by Bellingham from a smaller number, here as elsewhere saying the same thing, but in a far greater space. These conclusions are: "That indirect compression does not provoke the obliteration of the artery at the level of the compressed point; and second, that it acts on circumscribed aneurism by causing the deposit of active or fibrinous clots; consequently, it does not expose the patient to relapse, or inflammation of the sac, or the other inconveniences of passive clots." M. Broca very properly distinguishes diffused aneurism from circumscribed, for in it, as a moment's reflection would inform any one, the active clots could not form regularly, and passive clots would be formed. "But for that, indirect compression should not be considered inefficacious in the treatment of diffused aneurism, and as to the accidents by which it can be followed in such cases, they would be, at least, as frequent and as serious if the ligature had been preferred;" (p. 757.)

M. Broca gives a very careful and minute account of two cases of diffused aneurism, treated by himself, one in the wards of M. Malgaigne, the other in those of M. Nélaton. In the first, an enormous diffused aneurism of the tibio-peroneal artery, obliteration took place at the expiration of twenty days. But this obliteration was owing to the formation of passive clots, which, in place of condensing, becomes softer and more fluctuating, without being at all pulsating. This tumor was opened by a trocar, in order to relieve the pain, fever, and insomnia, it caused; black, softened, decomposed clots of blood, mixed with pus, were extracted. The patient was much relieved, when, thirteen days afterwards, the openings gave passage to an abundant arterial hemorrhage, this was relieved by direct compression. The next day, Malgaigne amputated the thigh, and eleven days after the operation the patient died of purulent infection. At the autopsy, the aneurism was found partially obliterated by a very consistent fibrinous mass, and partly by passive clots, which had softened, provoked suppurative inflammation, and, finally, had rendered amputation indispensable. All this has again and again been observed after the ligature.

In the second case, which is very interesting in many respects, a diffused aneurism of the popliteal region communicated with the knee-joint. Compression was tried with instruments, but the patient could not support them; owing to the condition of the joint, the limb was half flexed, and the instrument could not remain in place, without making it so tight as to be insupportable. With praiseworthy zeal, the students following M. Nélaton, offered to keep up uninterrupted digital compression upon the artery where it passes over the pubis; for ninety-four hours this was kept up, and when desisted from, there was no bruit-de-souffle, and the principal tumor was solid, and free from pulsation. A week afterwards, some very manifest movements of expansion, that had persisted at the sides of the knee, had disappeared. Three days afterwards, the tumor enlarged and became very painful; the fever was so very intense, and the general condition of the patient so alarming, that M. Nélaton amputated the thigh. On the seventh day of the amputation he died of purulent infection. At the autopsy, the aneurism was found to have become diffused consecutively, and while the primitive cavity was filled with active clots, the secondary, deprived of a sac, was filled with passive. These cases show, at all events, that indirect compression produced what the ligature would have produced. But that is all.

In treating of the *different modes* and *different proceedings* of indirect compression, of course, as the compression can be more or less forcible, it is possible to make several divisions, but M. Broca is content with two, total and partial, as the pulsations are totally suppressed or only diminished. As total compression (in the rare cases where it is performed), has the inconvenience of exposing to the same accidents as the ligature, as it is so painful that it might almost be called insupportable, as it endangers the healthy condition of the skin, and is often followed by œdematous and painful swelling of the limb,* total compression is a procedure so vicious, that it would not be worth while mentioning it, did it not enable M. Broca to make a regular proceeding of a certain manner of applying compression, which he calls *compression en deux temps.* Again and again patients have been treated in this way, and with success, but M. Broca says that this treatment was owing more to accidental circumstances than to the will of the surgeon. The best way will be, according to him, to imitate this treatment, and call it *compression en deux temps.* It would be unfair to say that he invented the proceeding, but in all probability he has invented its name. According to this proceeding, so soon as it is satisfactory that the aneurismal pocket is half obliterated by active clots, the pressure should be made total. He justly supposes that this mode offers some analogy with another, which is preferred by some surgeons, *gradual compression.*

Sometimes it is impossible to maintain the compression continuously, and it must be suspended for a time; from this, M. Broca makes two distinct modifications,—*intermittent* and *interrupted* compression. Intermittent compression is, when, on account of the pain, the pad must be loosened a few moments, or longer; it is interrupted, when, obliged to be abandoned for some time by accidental circumstances. These interruptions prolong the duration of the treatment, but the fibrinous layers that form persist.

Of all these proceedings, M. Broca prefers compression *en deux temps,* which he considers as certain and as inoffensive as partial compression, and as having, moreover, the advantage of shortening very much the duration of the treatment. This mode of applying compression, that is, of making the compression *total* after the lapse of a certain time, should be more often adopted than it is. In the Edinburgh Medical Journal, for 1856, there are two interesting cases reported, one by James Miller, the celebrated Professor of Surgery, the other by Dr. Johnstone. In both cases the compression, which was partial and interrupted, failed to cure the patients, though at first the tumor was much diminished in size. The ligature was afterwards applied with success. The whole history of these cases is exceeding instructive, but we can only call attention to these observations added by Mr. Miller; we do so on account of the opinions expressed

* Swelling of the limb is an objection made to the treatment by compression, but it is only applicable to total compression. Partial, generally dissipates any symptomatic œdema that previously existed.

therein in regard to the *collateral circulation*, which we have not met with elsewhere, and particularly on account of their bearing upon total compression, after partial has been continued for some time. As total compression acts exactly as the ligature upon the circulation, and is much less dangerous; and moreover, as it can be made supportable for a short time, which is all that is necessary in such cases, we would substitute it. "During the first weeks I had great hopes of obtaining a cure; the tumor became slowly harder, smaller, and less strong in pulsation. But after the third month, the impulse became decidedly more feeble, and with this change the hardening and diminution of the tumor stopped, and the tendency was rather the other way. I have no hesitation in attributing this result to the full establishment of most free collateral circulation, which the continued pressure had produced. And in every future case, were I to observe a similar change, I should at once supersede pressure by the ligature, satisfied that by such establishment of free collateral circulation, the success of pressure alone is rendered almost impossible, while at the same time delay of the ligature were most imprudent, lest, from the same cause, even its most abrupt and decided effects on the supply of blood might find some difficulty in silencing and shutting up the tumor." In a case very similar to those reported by the Edinburgh professor, and reported in the London Lancet, for February, 1856, Mr. Fergusson, instead of applying a ligature, applied Signorini's tourniquet on the superficial femoral. The total compression was kept up from 11½ A. M., to the next day at 10½, when it was removed. "All pulsation in the tumor had ceased." This has been observed repeatedly; we have never seen it elsewhere explained, however, than in the observations of Miller.

In regard to *the place where compression should be applied* (p. 792). M. Broca speaks at some length of pressure applied below the sac, that is, between it and the capillaries, plainly in order to show "that Vernet first applied it with a knowledge of what he was doing," and to call it the "*Method of Vernet.*" "In the few cases in which it has been applied, in one it was completely inefficacious; in two it caused the tumor to increase; in one it caused the tumor to burst; in two other cases one was somewhat improved, and one was cured. This last one, however, one of the external iliac, had been treated for 35 days previous by the method of Valsalva, and at the same time by direct compression."* According to M. Broca, these results are not such as to encourage any new trials. There is some reason for thinking, however, that by making the compression total, better results would be had. Many very competent surgeons believe that the rapid cures of aneurism, obtained by compression, are owing to the blocking,

* This is a very curious case, which M. Broca has very imperfectly reported (p. 797); he has taken it from Bellingham, loc cit. p. 61.

up of the distal portion of the artery by a clot; and total compression of the artery about the same place should produce the same result. Dr. Collis (Dublin Quarterly, November, 1855) says, that "when pressure is made on the distal side of an aneurism, we observe an increase of impulse for a few minutes. If the compression is *complete* and *firmly kept up* (sic), this momentary irritation subsides, and the aneurism pulsates with less force than before." What is more important, however, the same surgeon reports a case in which, by acting upon this principle, he cured a case of aneurism of the common femoral. When the pressure was incomplete, the tumor increased in size. "A double-clamp tourniquet was applied against the pubis, and not disturbed for thirty hours, at the end of which time, upon slackening it, all pulsation was found to have ceased in the tumor and in the artery as high as it had been traced before, namely, to the spot where the common iliac bifurcates. Here the artery was compressed close to the sac, and the nearest collaterals were the internal iliac on one side, and the profunda on the other; pressure was made between the sac and one of the next collateral branches. As soon as the circulation was completely arrested, there was a coagulum *en masse* (sic) above and below, of the blood between the collaterals. And to the coagulation above the tourniquet Collis attributes most probably the non-occurrence of relapse, or of suppuration of the sac." (Loc. cit. p. 319.)

In regard to the points to be chosen for compression, of course, they are those where the vessel is at once superficial, and near to an osseous surface capable of furnishing a *point d'appui;* and it is best not to approach too near to the tumor. The surgeon, without compromising the result, can go as far as he chooses from the seat of the aneurism. Two pads should be used alternately. This portion of M. Broca's book is rather extraordinary, and, as in many other places, he enters into such details, and lays down certain information in such a way, as to make one wonder what readers he wrote for; it is well for an author to remember, that though his knowledge may be in some points superior, nevertheless that his readers have reasoning faculties of their own.

As to whether it is proper to join direct compression on the tumor, M. Broca, in accordance with the views generally given, remarks very justly, that it opposes the obliteration of the sac, insomuch as to prevent the entrance of the blood, from which the fibrine is to be deposited, and that it is the best way of causing the failure of indirect compression. This is at the commencement of the treatment; when the obliteration is already advanced, he thinks that some advantages may be drawn from it, that it hastens the cure. General compression of the limb, he thinks still worse of, as it not only presses on the sac, but also on the collaterals, so as to interfere with their development.

As to the choice of the apparatus of compression, M. Broca enters into details of instrumental mechanics, which to him appear necessary, and at page 831, gives the representations of his *new apparatus for the compression of the femoral artery in every point of its length;* "without having imagined anything, and without having done anything, but to choose and combine the best and most simple of the known mechanisms, he believes that he has obtained an apparatus superior to all those employed to this day."

It is impossible to discuss critically this instrument, but there is one point that should not be passed over. The fundamental part of all the apparatuses is the compressing pad: this M. Broca says should be *small, convex*, and *hard*. The reasons he gives are theoretical, for it must "push aside the flesh, and avoid as much as possible the veins and satellite nerves of the artery." This is contrary to the experience of the Dublin surgeons, who, though they acknowledge it to be "incorrect theoretically," have found a broad, soft pad to answer best. (Bellingham, loc. cit. p. 184.) Digital compression, he acknowledges to be the most efficacious and least painful of all, the only objection to it being the difficulty of procuring the zealous assistance of a sufficient number of skilful persons. He says that it may be called an American proceeding, and gives the principal merit to Dr. Knight, of New Haven.

The instrument of M. Broca exerts an *elastic compression*, by means of vulcanized caoutchouc attached to the stem supporting the pad. Elastic compression was invented by M. Carte, and has enabled many patients to support an instrument, for it is much less painful than ordinary compression. Dr. Hutton says of the elastic pressure, "An easy mode of establishing its superiority exists, which any member of the profession can test upon himself. Let him place a screw-clamp upon the femoral at Scarpa's space, or at any other portion of the thigh, so as to stop pulsation in the vessel below. Let him try to walk across the room, he will find himself, as it were, riveted to the spot, totally unable to move. Let him next substitute for the clamp a circular compressor of M. Carte, and equally control circulation through the limb, and then try his powers of progression. Instead of inability to move, he will discover that he can proceed freely and walk with ease, the artery still remaining compressed, and no blood passing." It has also another advantage, in permitting the surgeon at a glance to recognize the degree of compression exerted on the artery.

§ VI. On the application of indirect compression, and the phenomena which result. A preparatory treatment, of a nature to diminish the mass of blood, and to render it more thick and more plastic, would favor, in his opinion, the action of compression, but as this method (compression) tests severely the resignation of a patient, he advises that the compressor be at once applied, reserving his patience for a more useful moment. As soon, however, as the pad is placed, certain measures drawn from the method of Valsalva must be used. The administration of digitalis should never be neglected, and in order to diminish the sensibility of the patient, preparations of opium should be given. He very prudently would not use ether and chloroform. This is not the advice given by the Dublin surgeons, who have been so successful. "The value of constitutional measures in these cases is not sufficiently insisted on, and yet I believe it will be found an important element in the treatment. If it were made a rule to institute a course of digitalis with low diet, in every instance, as an aid to compression, I apprehend, that mischief would frequently be done, and the cure of the aneurism retarded"—O'Farral says, at the same time giving proof of the truth of this opinion. (Dublin Quarterly for 1846, Vol. II.)

There is another point not to be passed over here: M. Broca says nothing about the position of the limb, attention to which tends greatly to facilitate the cure; thus, in brachial aneurism, the limb should be kept nearly in the perpendicular direction, and in femoral aneurism the foot much elevated. (See Crisp, loc. cit. p. 186.) O'Farral calls this one of the most valuable auxiliaries, favoring the return of the venous blood. (O'Farral, loc. cit. p. 383.)

In regard to the management of the apparatus, there is nothing to be remarked. It is natural to fear that the pads, compressing, always to a certain degree, the satellite vein of the artery, would so much interfere with the return of the blood as to produce œdematous tumefaction of the limb. This fear has very seldom been realized. M. Broca knows but of two cases. On the contrary, compression dissipates the œdema preexisting, caused by the aneurismal tumor interfering with the flow of blood in the adjacent vein. Compression interferes much more with the arterial circulation than with the venous, and the blood diminished in quantity finds sufficient ways to return to the heart.

In regard to the medium duration of the treatment, it has been, so far, below fifteen days; it is, therefore, less than that of the treatment by ligature. After the cure, the apparatus should be kept in place for several days, and thus with certainty, immediate relapse would be avoided, and, as to consecutive, M. Broca does not believe that compression properly executed has ever been followed by relapse; in those cases where it occurred, preferring with justice to accuse the surgeon (Bransby Cooper), rather than the method, for the insufficiency of the results. In those cases in which compression was not successful, and gave place to the ligature, more than seven-eighths were saved. When practised without this previous compression, which serves to dilate the collateral vessels, the mortality after ligature is one-third.

General results of the treatment of aneurisms by indirect compression, from 1842, until May, 1854. This is the date of the preparation of his table, in which there is a list of 163 cases. These cases are discussed by M. Broca with great ability, fairness, and spirit. Of this number, in 116 compression was efficacious, and in 47 it was abandoned as *intolerable*, or as *inefficacious*. The examples of intolerable compression are twelve, but in one case the compression was abandoned in two hours,* in another in five minutes, so that there are, in fact, but three cases, where it could have been intolerable, "*but the observations are, perhaps, more intolerable still;*" (p. 867.) In one of them, published in the Monthly Journal, devoted to Mr. Syme, and, consequently, very unfriendly to compression, Mr. Bergin is the operator, and his proceeding, as given by M. Broca, is exceedingly amusing; (p. 868.) In the other two cases there is no detail, but the surgeon says, he had only *imperfect instruments* at his disposal.

The examples of compression reputed inefficacious, are thirty-five. In thirteen, compression was certainly badly applied. In fine, in only eleven cases will M. Broca acknowledge that it was inefficacious, and in these the absence of active coagulation he assigns to a peculiar disposition of the blood, a want of plasticity of an unknown nature. The mortality does not reach five per cent. Only six deaths can be imputed to the treatment; four consecutive to compression alone, and two to ulterior ligature. Finally, of the four produced by compression, of only one can the method be accused, a case in which erysipelas was developed under the pad. The others died from the obliteration of the aneurism, and would have died if this had been effected by a ligature.

After what precedes, M. Broca properly judges that it would be superfluous to establish a long parallel between compression and ligature, and makes a few, but very forcible remarks on this point.

Chapter XX. On the choice of methods according to particular cases. (p. 877.) This is a very able chapter, in which, after remarking upon the folly of establishing a general parallel between methods, he passes in review particular cases, in order to compare them with some advantage. The seat of the tumor, its nature, its size, the state of the parts around it, the extent of its complications, the imminence of the danger, are so many eventualities that complicate the problem offered the surgeon. In the first place arterial aneurisms must be distinguished from arterio-venous.

Aneurisms of the thoracic aorta can only be treated by the method of Valsalva. Those of the brachio-cephalic trunk, by the method of Valsalva,

* Mr. Lawrence, in American Medical Journal for October, 1851, from Lancet for July. By means of the usual clamp-shaped instrument, the pulsations of the tumor were completely arrested. "The patient, however, stated that the pressure gave him intense pain, and that he should probably not be able to endure the compression long. Nor did his feelings deceive him, for he could endure the apparatus but two hours."

aided by refrigeration. If that would not do, the carotid should be tied, and afterwards the subclavian, if the first ligature be unsuccessful. Aneurisms of the inferior extremity of the carotid by Brasdor's proceeding, and of the superior, by the method of Anel. The common carotid should also be tied when the internal carotid is affected, or the external very near its origin. In aneurisms of the ophthalmic artery, on account of the gravity of ligature of the primitive or internal carotid, and of the facility with which the circle of Willis brings back the blood, it would be prudent to make some attempts with galvano-puncture, and then with the perchloride of iron. Aneurisms of the external carotid are divided into two categories: those occupying the face and upper part of the neck, and those situated on the cranium; the first, when small, are to be treated by coagulating injections and galvano-puncture; when of a certain volume, however, Anel's method can alone be counted upon. When on the skull, he would commence by an application of the perchloride of iron, by the endermic method, and if that did not do, he would inject it into the sac.

Aneurism of the arteries of the upper extremity.—In treatment of aneurism of the subclavian artery, he would try the method of Valsalva; if it still increased, galvano-puncture, and as a last resource, would tie the artery beyond the tumor. In aneurism of the axillary artery, the method of Anel finds all its superiority; it is on the subclavian, outside of the scaleni, that the ligature should be applied. Aneurisms of the upper third of the humeral should be treated by a ligature placed on the artery in the armpit. When below that, indirect compression can and should be employed. Traumatic aneurism at the elbow merits particular mention. When small and recent, the treatment first employed should be direct compression, for which forced flexion of the arm, as advised by Malgaigne, is sufficient. When it is of long standing, direct compression will probably fail, indirect also, and the ligature must be had recourse to.

Aneurism of the abdominal aorta and its visceral branches, require the method of Valsalva, also those of the primitive iliac, for the method of Brasdor has never succeeded in the treatment of sub-diaphragmatic arteries, and to apply the method of Anel, the aorta must be tied, an operation that should be banished from surgery. If an aneurism of the internal iliac was diagnosticated, the primitive iliac would have to be tied, but M. Broca knows no instance of aneurism developed *in the pelvis* on the branches of this artery. Aneurisms of the buttocks are not rare; when a sufficient interval exists, the artery must be tied after it has issued from the pelvis; when not, the primitive iliac must be tied. Aneurisms of the external iliac demand the application of the method of Anel; when the aneurism mounts so high that it is not possible to tie the external iliac above the tumor, the primitive iliac must be tied. As this is a very dangerous operation, a moderate direct compression should be exercised on the tumor, through the walls of the abdomen, the femoral should be compressed in the groin, and absolute repose, diet, digitalis, and bloodlettings should be added. This pressure in the groin, from what he says before, has been so very unfortunate, that it is extraordinary that he should recommend it. Inguinal aneurism must be treated by ligature of the external iliac, and all other arterial aneurisms of the inferior extremity by indirect compression. If compression fail, ligature must be had recourse to. According to M. Broca, the true point selected for ligature in aneurisms of the leg, ought to be the inferior extremity of the femoral artery, and not at the angle of the inguinal triangle, as many surgeons advise. For those of the foot and of the instep, for those that exist behind the internal malleolus, or that occupy the inferior third of the anterior tibial, the trunk itself of the aneurismatic artery should be tied. For arteries placed higher up, the ligature of the femoral seems to him preferable to that of the popliteal, and to that of the deep-seated arteries of the leg.

Different natural or accidental circumstances may modify these therapeutical indications which M. Broca passes in review in a separate paragraph. We can hardly think that it is necessary to say that diffused aneurisms should never be treated by the method of Brasdor. Aneurisms that have burst into the muscles, and with bloody infiltration of the whole limb, demand amputation; aneurisms that have opened externally with hemorrhage, require ligature, indirect compression, incision of the sac and tamponnement with perchloride of iron.

Complicated with inflammation, aneurism must be actively treated. It is capable of leading to their spontaneous cure, but it is accompanied with dangers so numerous that it is indispensable to combat it. When the inflammation is but slight, repose, indirect compression, and the application of a bladder filled with ice, are generally sufficient to arrest it. If the seat of the aneurism does not permit of compression, the ligature must be practised without delay. When more considerable, if the tumor be still pulsating, the surgeon must tie the artery above, but if the pulsations are arrested the question is difficult. If the inflammation has not lasted long enough to obliterate the artery satisfactorily, the ligature should at once be used; if it has, he can wait, but in every case the opening of the tumor must be delayed as long as possible.

M. Broca knows of no case of gangrene of the limb complicating aneurism before the total obliteration of the aneurism. As a general rule, the surgeon should wait until the gangrene is limited; when he cannot, on account of the general symptoms, he must amputate above the tumor. Aneurisms communicating with joints, sometimes demand amputation; and also those that have caused deep and extended erosion of the skeleton. When an external aneurism is complicated with an internal, every operation must be desisted from.

The method of Valsalva, the method of Anel, and indirect compression, have no hold on arterio-venous aneurisms. The opening of the sac, double ligature, direct compression, galvano-puncture, and coagulating injections, can only be counted upon. As the greater number of these aneurisms remain stationary throughout life, and give rise to no dangers, it is only when they increase that an operation should be thought of; and the surgeon should always commence by direct compression. Arterio-venous aneurisms of the aorta, of the iliacs, of the carotid, subclavian, and axillary arteries cannot be treated curatively. Of the thigh and groin, the only treatment is direct compression, and when this method is not applicable, he advises them to be left in repose, as also of the popliteal region and thick parts of the leg. Of the foot, direct compression may be tried, and if it fail, some attempts may be made with galvano-puncture and coagulating injections. Analogous principles should guide the surgeon in the treatment of arterio-venous aneurisms of the upper extremity; but M. Broca stops a moment to consider those of the elbow, which are much more common. As here the aneurism is superficial, generally not large, situated on an artery of medium calibre, and not far removed from an osseous surface on which it is easy to compress it, the conditions are all favorable to direct compression. M. Broca is satisfied that by patience, the greater number of varicose aneurisms can thus be cured. If it fail, a bloody operation is too serious to think of; galvano-puncture could be tried, and if that did no good, perchloride of iron could be injected. The method of double ligature should be reserved for the very exceptional cases where the tumor makes alarming progress.

M. Broca has attached to his work two tables, containing 215 observations of aneurism treated by indirect compression; the first, of 62, is composed of facts anterior to the Irish period; the second, of 163, all from thence to May, 1854.

The index is the best we have ever seen in a French medical work.

